# Consequences of COVID-19 Lockdown on Children and Their Pets: Dangerous Increase of Dog Bites among the Paediatric Population

**DOI:** 10.3390/children8080620

**Published:** 2021-07-22

**Authors:** Giovanni Parente, Tommaso Gargano, Marco Di Mitri, Sara Cravano, Eduje Thomas, Marzia Vastano, Michela Maffi, Michele Libri, Mario Lima

**Affiliations:** Paediatric Surgery Department, IRCCS Sant’Orsola-Malpighi University Hospital, 40138 Bologna, Italy; tommaso.gargano2@unibo.it (T.G.); marcodimitri14@gmail.com (M.D.M.); sara-cravano@libero.it (S.C.); edu.thomas92@gmail.com (E.T.); marzia.vastano@icloud.com (M.V.); michela.maffi@libero.it (M.M.); mlibri31@yahoo.it (M.L.); mario.lima@unibo.it (M.L.)

**Keywords:** COVID-19, lockdown, dog bite, injury, trauma

## Abstract

Background: The SARS-CoV-2 pandemic has not only put our national health systems to the test, but it also notably hit the economy, the society and the psychology of the people. Our pets have been subjected to the pandemic related stress too. The aim of the study was to evaluate whether the stress induced on domestic dogs resulted in an increase of dog bites in the paediatric population. Methods: A retrospective study was conducted on all patients admitted to our paediatric emergency department for dog bite from January 2014 and December 2020. We compared the total mean dog bites of the years 2014–2019 and the mean number per single month with the respective 2020 data. The bites were divided between bites from family dogs and bites from stranger dogs. Continuous data were analysed using single sample *t* test while categorical values with chi-squared test, considering statistically significant a *p* value < 0.05. Results: From January 2014 to December 2019, we recorded a mean of 41 ± 5.9 dog bites (range: 30–46) of which a mean 13 ± 2.6 (range: 10–17) were due to family dogs (32%) and a mean of 28 ± 2.4 (range: 25–31) were due to stranger dogs (68%); the male-to-female ratio was 3:2 and 43% of the injuries concerned the head and face, 26% the lower limbs, 25% the upper limbs, 3% the genitalia and 3% the torso. From January 2020 to December 2020, 30 children were admitted for dog bites: 22 were from family dogs (73%) and 8 from stranger dogs (27%); the male-to-female ratio was 14:11 and 72% of the injuries concerned the head and face, 16% the upper limbs, 8% the lower limbs and 4% the torso. The 2020 data’s higher number of family dog bites compared with the mean of those in the 2014–2019 period was statistically significant (*p* < 0.01) such as the 2020 data’s lower number of stranger dog bites when compared with the mean number of stranger dog bites in the 2014–2019 period (*p* < 0.01). Between 2014 and 2019, a mean of 9 ± 2 (range: 6–12) of the wounds needed to be sutured (22%), while 32 ± 3 (range: 28–35) wounds were discharged after application of Steri Strips (78%). On the other hand, in 2020, 21 wounds needed to be sutured (70%), and 9 received just Steri Strips application (41%). The frequency distribution of the treatments required (stitches vs. Steri Strips) between the 2014 to 2019 period and the 2020 period was statistically significant (*p* < 0.0001). Conclusions: The number of family dog bites in children increased in 2020, especially during the lockdown period. Paediatricians should pay a lot of attention now more than ever to educate parents on the importance of always supervising children who are playing with dogs.

## 1. Introduction

From late 2019, the entire world began facing the COVID-19 pandemic, with Italy as one of the most affected countries [1]: the pandemic has not only put Italy to the test by saturating intensive care units all across the country, but it also had and will continue to have serious repercussions on our economy, society and psychology [1,2].

Along with wearing masks, practicing hand hygiene and social distancing, one of the main measures adopted all around the world against SARS-CoV-2 spread was the confinement, also known as “lockdown”. The “stay-at-home” requirement, although effective in confronting the pandemic [3], also brought with it, as mentioned above, inevitable negative consequences on people’s psychological health, as seen in the increased incidence of anxiety and depression [4].

Pets during the pandemic era are reported in the literature as having an important role in mitigating loneliness and safeguarding their owners’ mental health. This emotional buffering effect is explained by the opportunities that pets give people to touch and feel another living creature in the house, and moreover, the necessity of having to take dogs out for a walk gives the dog owner an opportunity to continue having social interactions with other people in spite of lockdown [5,6,7,8,9].

Nevertheless, we should not only analyse the pandemic and the relationship between owners and pets only from the human point of view, but researchers should also investigate how pets themselves experienced these peculiar months.

Research studies have claimed a worsening of pets’ health as well as an increase in multiple behavioural problems, especially in dogs, including excessive vocalization, urination/defecation at home, destructiveness, aggressiveness towards family members or other animals in the house and fear of loud or sudden noises, either appearing for the first time or worsening in cases in which such behaviours were already present—all due to the increase of dogs’ anxiety and stress related to the lockdown and the limitations it imposed on their daily routine [10,11].

Due their direct experience in emergency departments and the vast literature on the subject, paediatricians and paediatric surgeons already knew that children, in particular toddlers and those in preschool, were at higher risk of being bitten due to their impulsive and often incorrect approach to animals [12,13,14,15,16], Additionally, some authors tried to identify risk factors in order to prevent these episodes from happening [17,18,19].

Considering concerns in literature about dogs’ negative behavioural changes during lockdown, along with their higher exposure to children in the home context, the aim of the study was to evaluate whether the circumstances of the lockdown resulted in an increase in dog bites in the paediatric population.

## 2. Materials and Methods

### 2.1. Study Design and Population

A retrospective study was conducted using data from January 2014 to December 2020 on patients seen in the paediatric emergency department of IRCCS Sant’Orsola-Malpighi, a third-level hospital of Bologna (Italy), a city with more than 1.02 million inhabitants in the metro area, not to mention those in the province. All records of the emergency department were reviewed.

General characteristics of the population were taken into account, such as sex, mean age of the patients and the anatomic site of injury, classified as head and face, upper and lower limbs, torso and genitalia.

Patients with injuries that required just dressing applications or that needed to be sutured without general anaesthesia were sent home after treatment and medications, while children that needed suture under general anaesthesia were admitted into our paediatric surgery unit and discharged the day after.

### 2.2. Statistical Analysis

We compared the mean total dog bites per year between 2014 and 2019 and their single month mean number with respective data from 2020.

The injuries were divided in those due to family dogs and those due to stranger dogs.

The Shapiro–Wilk test was performed to verify a normal distribution of the number of bites (total number, family dog bites and stranger dog bites) of the 2014–2019 group.

Single sample t test was performed to compare 2020 data with the mean values of the 2014–2019 period.

Proportions were analysed with Yate’s chi-squared test.

We set α = 0.05; therefore, a *p*-value < 0.05 was considered statistically significant.

Data regarding the age of patients and the number of bites that occurred (total, familiar and stranger dogs) are reported as mean ± standard deviation.

## 3. Results

**Figure 1:**
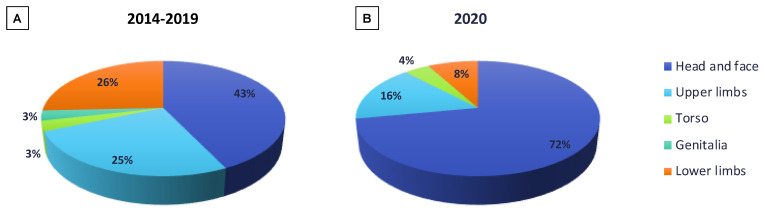
Anatomical distribution of the sites of bites during the 2014–2019 period (**A**) and 2020 (**B**).

From January 2014 to December 2019, we recorded a mean of 41.0 ± 5.9 dog bites/year (range: 30–46) of which a mean 13.0 ± 2.6 (range: 10–17) were due to family dogs (32%) and a mean of 28.0 ± 2.4 (range: 25–31) were due to stranger dogs (76%); the male-to-female ratio was 3:2 and the mean age of patients was 6.4 ± 3.5 years (range: 8 months–13 years); 43% of the injuries concerned the head and face, 26% the lower limbs, 25% the upper limbs, 3% the genitalia and 3% the torso (Figure 1A).

From January 2020 to December 2020, 30 children were admitted for dog bites: 22 were from family dogs (73%) and 8 from stranger dogs (27%); the male-to-female ratio was 4:1 and the mean age was 5.2 ± 3.7 years (range: 9 moths–13 years); 72% of the injuries concerned the head and face, 16% the upper limbs, 8% the lower limbs and 4% the torso (Figure 1B).

A Shapiro–Wilk test showed a normal distribution of the data from the 2014–2020 group (total number of bites/year: *p* = 0.15; number of family dog bites/year: *p* = 0.94; number of stranger dog bites/year: *p* = 0.87).

The 2020 data’s higher number of family dog bites compared with the mean number of family dog bites in the 2014–2019 period was statistically significant (t(df: 5, two-tailed) = −8.45, *p* < 0.01) as long as the 2020 data’s lower number of stranger dog bites was compared with the mean number of stranger dog bites in the 2014–2019 period (t(df: 5, two-tailed) = 20.70, *p* < 0.01).

Between 2014 and 2019, a mean of 9 ± 2 (range: 6–12) of the wounds needed to be sutured (22%), while 32 ± 3 (range: 28–35) wounds were discharged after application of Steri Strips (78%).

On the other hand, in 2020, 21 wounds needed to be sutured (70%), and 9 received Steri Strips application (30%).

The frequency distribution of the treatments required (stitches vs. Steri Strips) between the 2014 and 2019 period and the 2020 period was statistically significant (χ^2^ (df: 1, N: 71) = 14.48, *p* < 0.01)).

Our data illustrated a statistically significant increase in dog bites from familiar dogs rather than from stranger dogs in 2020 (COVID-19 outbreak) if compared with 2014–2019; moreover, the number of bites that required sutures were significantly higher in 2020 as an indirect sign of greater violence in the act of biting. Taken together, the results are indirectly suggestive of familiar dogs’ higher level of anxiety, stress and behavioural disorders resulting in more aggressiveness towards children.

Data reported above are presented in Table 1.

## 4. Discussion

The SARS-CoV-2 pandemic has severely affected the entire world population.

Health systems around the world have been severely tested, but Coronavirus was not just a hospital affair. Moreover, the consequences on mental health of the world population will be equally serious, with increases in isolation, depression and anxiety disorders [4].

Even if they are rarely mentioned, we should not forget that our pets are also included in this anomalous situation.

The main public health measure to limit the virus spread imposed by almost all the countries most severely affected by Coronavirus was the “lockdown”: the order to stay at home except for fundamental needs [3].

This meant that entire families remained at home, increasing the sharing of spaces with their pets and their greater exposure to children who interact with the animals while not always being supervised by adults.

Even though we are aware that dog owners experienced advantages in having a dog by their side, in terms of feeling of loneliness attenuation and, therefore, protection from the development of mental disorders such as depression [5], studies that present only positive outcomes of dog ownership have been targeted on the adult population or, at least, adolescents [5,6,7,8,9].

Bowen et al. studied how the confinement affected the human–animal bond during the Spanish lockdown from both the human and the canine point of view [11]. Their results showed that, as regards humans, the owners of pets declared that during the confinement the closeness to their animal helped them in overcoming isolation and sadness. On the contrary, the confinement seemed to negatively affect the quality of life of dogs: the commonest behavioural problems that quickly worsened during lockdown were annoying or excessive vocalization and fear of loud or unexpected noises.

Moreover, the pets’ owners also reported a higher incidence of the following behavioural problems: attention-seeking, irritability and a more excitable tendency, frustration and stress [10,11].

The main explanation for the behavioural worsening of dog behaviour during lockdown is surely the reduction in the number and duration of walks per day, forcing the animals to spend most of their time in a closed home environment. Spending time together in activities such as walking is important in establishing a stable bond within the owners and their dogs and, on the other hand, it is an opportunity for the dog to express freely their “dog-like” characteristics, such as sniffing, greeting and playing with other dogs [10,11]. This last aspect was one of the activities most affected by lockdown: the constraint of going out only for essential needs and the impossibility venturing beyond the immediate neighbourhood sometimes made it impossible for owners to bring their dogs to large open spaces or dedicated parks, restricting dogs to only on-lead walks on the streets. Moreover, social distancing made isolated walking routes preferred, preventing interactions between dogs.

All of the facts mentioned above made walks boring for the dogs, and the restrictions on their freedom and interactions was seemingly expressed in frustration and nervousness, as reported by Holland et al. [10].

An aspect that should not be underestimated is that during confinement caregivers had the additional stress of children staying at home 24 h a day, 7 days a week, for months without or with less possibility of out-of-home activities (school, playdates, parks, etc.), sometimes accompanied by concern and anxiety resulting from the loss of jobs where home working was not possible, such as those employed in restaurants, tourism, etc. In these situations, dogs may experience a phenomenon that has been described as contracting an “emotional contagion”: a state in which companion dogs mirror the emotions and stress levels of their human caregivers [11,20].

It is in this frustrating, anxious and nervous scenario for our dogs that we focus the attention on the relationship with children.

It is known that children are at high risk of dog bites, especially children under the age of 5, and that because of their short stature these events often result in face and neck injuries, with potential functional and aesthetic repercussions [12,13,14,15,16,17,18,19].

One reason children under the age of 5 are bitten by familiar dogs is because children unknowingly provoke the animals (high-pitched voices, sudden movements, inappropriate interaction) [19]. Moreover, young children are unlikely to recognize the emotions or behavioural signals of dogs provoked by unwanted child behaviours [21].

For example, in one study of the interactions of children aged 2–5 years with the family dog, children frequently initiated risky interactions with the pets by pulling dogs’ tails, hair or paws, and on nearly one-third of such occasions, such dogs bit or attempted to bite the children [22]. In addition, placing young children at heightened risk of dog bites are children’s natural tendencies to be active, loud and excitable, and to be protective of their property. Running and screaming can scare and anger dogs. Child–dog conflicts over toys and other property can lead to biting incidents [23]. Further, although dogs have some ability to identify familiar faces [24], dogs obviously do not have the cognitive skills of humans and are unable to recognize that children may be more provocative or animated than adults.

With these premises, we decided to investigate whether the lockdown has favoured the increase in the number of dog bites in children.

Dixon et al. are the first and only researchers in literature who described an increase of dog bites from March to May 2020 during the confinement in the USA, but they analysed just the first wave of the Coronavirus outbreak [25].

With our data, we show the entire nature of 2020 dog bites in Bologna, an Italian city and one of the countries with the most severe confinement regulation.

**Figure 2:**
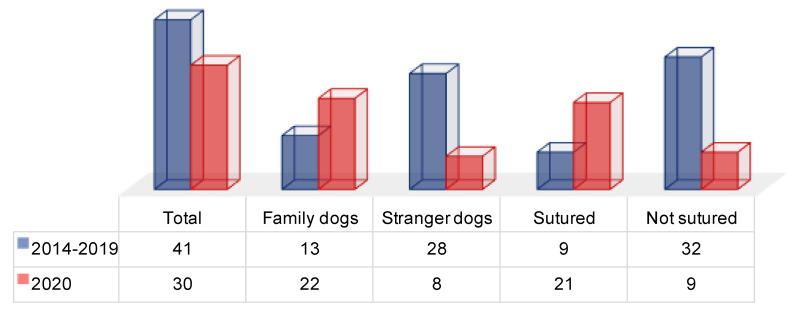
Number of dog bites and sutured injuries (the numbers of 2014–2019 are average values).

Considering the average number of dog bites in the period between 2014 and 2019 and the number of bites that occurred in 2020 (41 vs. 22), it would seem that the number of these events has decreased (Figure 2).

Actually, these data must be read with extreme attention: the number of dog bites in 2020 would seem to be lower only because the number of stranger dog bites was drastically reduced by 71.4% (*p* < 0.01). Stranger dog bites typically occur on the street or at the parks and playgrounds, places children have been less exposed to due to confinement, which explains the decline in the number of those previously exposed in the total number per year.

The worrying aspect, however, is the notable increase in bites by family dogs, which increased by 69.2% compared with the 2014–2019 average (*p* < 0.01).

Perhaps, even more alarming is the significant (*p* < 0.01) increase in the number of wounds that needed to be sutured, an expression of greater violence exerted by the dogs during the act of biting, not to mention the higher incidence of bites that injured the face (72% in 2020 vs. 43% between 2014 and 2019) of the patients resulting in inaesthetic scars.

**Figure 3:**
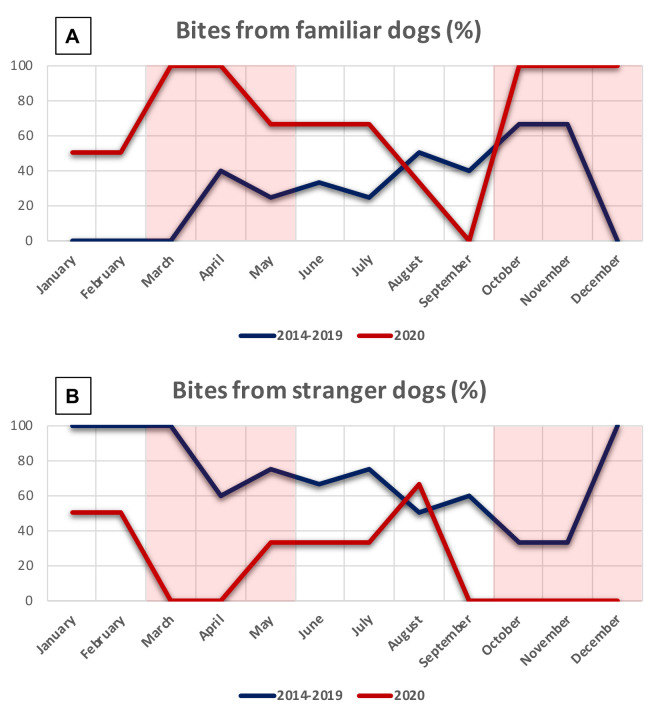
Relative numbers of dog bites/months. The red areas represent the two Italian lockdown periods. (**A**) Bites from familiar dogs. (**B**) Bites from stranger dogs.

Analysing the number of bites per month, as shown in Figure 3A, it is possible to see how two of the highest peaks of incidence of bites from family dogs are located during the two lockdowns Italy underwent in 2020; equally remarkable is that, even if decreasing, the relative number of bites per month from dogs to their paediatric owner remains often higher compared with those of 2014–2019.

The higher 2020 numbers of bites between the two lockdown periods could be easily explained as follows.

Holland et al. demonstrated in their paper that spending extra time with our dog(s) may negatively impact on the animal’s future ability to cope when left alone [10]. In fact, when movement restrictions are dropped and society returns to routine life, the dogs may continue to experience the stress and frustration derived by their reduced autonomy, becoming more attention-seeking and sometimes aggressive [11].

In Figure 3B is shown the lower relative number of bites per month from stranger dogs that had a peak during summer 2020, when the restrictions were abolished (except for face masks and social distancing).

What the data of the present study tell us, given the higher risk factors described above, is that during lockdown paediatricians and healthcare providers in general have to increase their efforts to promote prevention in order to reduce the number of dog bites.

Therefore, paediatricians have the duty to educate parents of children, especially pre-school and school-age children who have higher risk of dog bites (confirmed by our data too), reminding them that the most important way to prevent dog bites is to always supervise infants and children when they are near a dog [18,25,26,27] and to teach their children how to have proper interactions with a dog.

**Figure 4:**
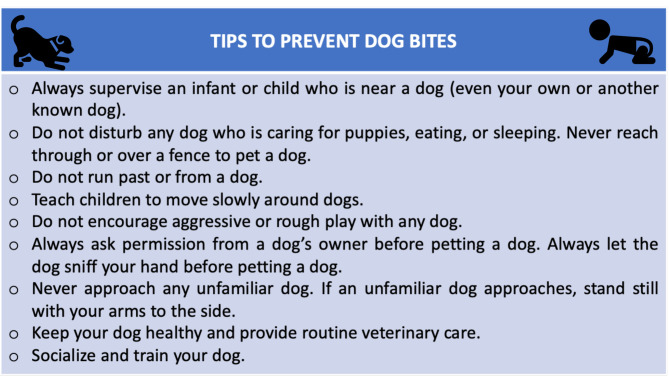
Tips to prevent dog bites.

For this purpose, the authors present here a list of tips to avoid dog bites that was disseminated by the American Academy of Pediatrics (Figure 4) [26] and already mentioned by Dixon et al., who suggests widely sharing this table with parents of children and owners of dogs [25].

Finally, the authors reinforce the importance of dog bite prevention programs based on obedience training, controls on high-risk breeds and the use of leashes. Combining child-oriented interventions with program and policy targeting dogs and their owners may be the best strategy to reduce dog bite incidence even under peculiar circumstances such as lockdowns [28].

Our study is the first that tried to correlate a suspicious increase in the number of dog bites in the paediatric population during the COVID-19 outbreak with the effect of confinement on pets. The discussion, summary and explanation of the concerns reported in the literature about behavioural changes in pets during lockdowns could help make paediatricians aware of the peculiar and dangerous situation and consequently could advise parents on the need for more careful supervision of interactions between their children and dogs.

On the other hand, the authors are aware of the limitations of study. The first is its retrospective nature, but it was difficult to imagine a prospective study since the pandemic was of course unexpected. The low number of patients enrolled is another drawback of the present paper; multicentre studies are needed to overcome this limitation.

## 5. Conclusions

In the literature there is a growing concern about the negative behavioural changes occurring in pets during confinement for the COVID-19 outbreak, and the authors wondered if this resulted in an increased number of dog bites in the paediatric population.

Data of the present study confirmed our hypothesis, showing an increase in the number and severity of bites, especially from familiar dogs. This could be an indirect sign of pets’ higher level of stress and anxiety due to confinement.

Health care providers and paediatricians have a duty to focus attention on this undesirable and dangerous event with parents, especially parents of young children, particularly since SARS-CoV-2 requires restrictive measures such as lockdowns.

This topic is of particular importance, and awareness is required between paediatricians and paediatric surgeons, reminding parents that prevention is the only way to reverse a phenomenon that risks becoming exponential.

## Figures and Tables

**Table 1:** Summary of the demographic data of the population along with *p*-values of the variables studied. The 2014–2019 are reported as mean yearly values ± standard deviation and ranges are reported between round brackets. m: months; y: years.

	2014–2019	2020	
Number of children bitten	41.0 ± 5.9 (30–46)	30	*p* < 0.01
Male:female ratio	3:2	4:1	
Mean age of children bitten	6.4 ± 3.5 y (8 m–13 y)	5.2 ± 3.7 y (9 m–13 y)	
Bites from familiar dogs	13 ± 2.6 (10–17)	22	*p* < 0.01
Bites from foreign dogs	28 ± 2.4 (25–31)	8	*p* < 0.01
Sutured vs. not sutured injuries	9 ± 2 (6–12) vs. 32 ± 3 (28–35)	21 vs. 9	*p* < 0.01

## Data Availability

The data presented in this study are available on request from the corresponding author. The data are not publicly available due to privacy restrictions.
